# A machine learning PROGRAM to identify COVID-19 and other diseases from hematology data

**DOI:** 10.2144/fsoa-2020-0207

**Published:** 2021-06-12

**Authors:** Patrick A Gladding, Zina Ayar, Kevin Smith, Prashant Patel, Julia Pearce, Shalini Puwakdandawa, Dianne Tarrant, Jon Atkinson, Elizabeth McChlery, Merit Hanna, Nick Gow, Hasan Bhally, Kerry Read, Prageeth Jayathissa, Jonathan Wallace, Sam Norton, Nick Kasabov, Cristian S Calude, Deborah Steel, Colin Mckenzie

**Affiliations:** 1 Department of Cardiology, Waitematā District Health Board, Auckland, New Zealand; 2 Clinical Information Services, Waitematā District Health Board, Auckland, New Zealand; 3 Clinical laboratory, Waitematā District Health Board, Auckland, New Zealand; 4 Department of Hematology, Waitematā District Health Board, Auckland, New Zealand; 5 Department of Infectious diseases, Waitematā District Health Board, Auckland, New Zealand; 6 Institute for Innovation & Improvement (i3), Waitematā District Health Board, Auckland, New Zealand; 7 Nanix Ltd, Dunedin, New Zealand; 8 Knowledge Engineering & Discovery Research Institute (KEDRI), Auckland University of Technology, Auckland, New Zealand; 9 School of Computer Science, University of Auckland, Auckland, New Zealand; 10 Sysmex New Zealand Ltd, Auckland, New Zealand

**Keywords:** biological age, COVID-19, full blood count, heart failure, hematology, machine learning, pneumonia

## Abstract

**Aim:**

We propose a method for screening full blood count metadata for evidence of communicable and noncommunicable diseases using machine learning (ML).

**Materials & methods:**

High dimensional hematology metadata was extracted over an 11-month period from Sysmex hematology analyzers from 43,761 patients. Predictive models for age, sex and individuality were developed to demonstrate the personalized nature of hematology data. Both numeric and raw flow cytometry data were used for both supervised and unsupervised ML to predict the presence of pneumonia, urinary tract infection and COVID-19. Heart failure was used as an objective to prove method generalizability.

**Results:**

Chronological age was predicted by a deep neural network with R^2^: 0.59; mean absolute error: 12; sex with AUROC: 0.83, phi: 0.47; individuality with 99.7% accuracy, phi: 0.97; pneumonia with AUROC: 0.74, sensitivity 58%, specificity 79%, 95% CI: 0.73–0.75, p < 0.0001; urinary tract infection AUROC: 0.68, sensitivity 52%, specificity 79%, 95% CI: 0.67–0.68, p < 0.0001; COVID-19 AUROC: 0.8, sensitivity 82%, specificity 75%, 95% CI: 0.79–0.8, p = 0.0006; and heart failure area under the receiver operator curve (AUROC): 0.78, sensitivity 72%, specificity 72%, 95% CI: 0.77–0.78; p < 0.0001.

**Conclusion:**

ML applied to hematology data could predict communicable and noncommunicable diseases, both at local and global levels.

## Lay abstract

Identifying and monitoring an infectious disease within a community requires sampling of both symptomatic and asymptomatic individuals. Hematology data from a full blood count contains significantly more information than used clinically. We applied machine learning to hematology data to predict the presence of both communicable and noncommunicable diseases.

## Background

COVID-19 has become a worldwide global threat to public health. The principle methods by which the infection can be controlled are through rapid identification and isolation of cases, contact tracing and strict infection control measures. The initial outbreak occurred in Wuhan, China and the early experiences from the region have been widely publicized, as well as scrutinized in medical journals [1,2]. COVID-19 can cause severe respiratory symptoms [3], cardiac injury and is associated with relatively high ICU admission rates and mortality. Severe acute respiratory syndrome coronavirus 1 (SARS-CoV-1) caused significant long term sequelae such as impaired glucose tolerance, pulmonary fibrosis and lipid metabolism [4]. Although numerous blood changes occur in response to COVID-19, the current gold standard to confirm the presence of SARS-CoV-2 infection is a PCR-based nucleic acid assay based on the original virus sequence data published within days of the first public notification of the outbreak [5]. Maintaining adequate PCR testing rates in the community has been challenging and alternative methods for predicting the presence of COVID-19 infection would be useful.

Machine learning (ML) holds significant promise in the prediction of COVID-19 when applied to hematological and standard laboratory testing [6–14]. This has advantages to a criteria-based PCR testing as hematology and laboratory testing is generally ubiquitous, and is performed on a wide scale across the population on a daily basis, quickly and at low cost. Although, a screening test requires a high level of sensitivity and specificity, an Oxford group has reported an AUROC as high as 0.94 and specificity of 96%, using ML applied to combinations of standard laboratory and clinical parameters [15]. Other independent efforts have also demonstrated a similar ability at identifying COVID-19 from routine blood testing augmented by ML [16–18].

The immunologic and hematologic changes associated with COVID-19 are profound and have been readily demonstrated with numerous studies using flow cytometry [19–23]. Hematology analyzers, present in many clinical laboratories, utilize a flow cytometry method which quantifies the intensity of fluorescence emitted from cells to produce a multitude of results, most of which are discarded before being delivered as a full blood count (FBC). A simple decision tree has been applied to hematology data to discriminate common causes of febrile illness in South East Asia [24]. Similarly, artificial neural networks have been applied to similar data to identify acute promyelocytic leukemia [25]. These data are readily available and performed in high volume, with over 40,000 FBCs done every day in New Zealand.

Prior to the SARS-CoV-2 pandemic, we activated a number of advanced hematology parameters on XN-1000 and XN-3000 analyzers (Sysmex, Kobe, Japan) present at Waitakere and North Shore Hospital, respectively, giving us a unique insight into the period leading up to and through both of New Zealand's lockdown periods. Due to the excellent public health response, government communication and willingness of the New Zealand public to adhere to public health messages the number of COVID-19 patients in New Zealand has been low. We describe here the hematology and laboratory profiles of COVID-19 patients in Waitematā, the largest District Health Board in New Zealand. We also aimed to demonstrate the power of ML not only applied to COVID-19 but also noncommunicable disease, using the example of heart failure. Finally, we explore the possibility of using this pattern recognition of global responses and monitoring (PROGRAM) as part of a national surveillance program for future pandemic management.

## Materials & methods

### Data collection

This study took the form of a retrospective observational nested cohort and case–control study. Ethics approval was obtained locally from the Research and Knowledge Centre at Waitematā District Health Board in collaboration with the Institute for Innovation + Improvement (i3), and from the regional Health and Disability Ethics Committee (HDEC) (20/CEN/162). Informed consent was waived, as the research was observational and used secondary data. Hematology raw data were collected between 1 July 2019 and 8 June 2020 from the Information Process Unit (IPU) connected to both a XN-1000 (Sysmex) at Waitakere Hospital and XN-3000 (Sysmex) at North Shore Hospital. This included hospital data from both inpatients and outpatients within Waitematā District Health Board’s (WDHB) catchment. Data were downloaded from the IPU in a comma separated value (CSV) data format, containing both basic and advanced hematology parameters. Flow cytometry standard (FCS) files were also downloaded only for SARS-CoV-2 positive patients with randomly selected matched number of controls. Standard biochemical laboratory data were extracted from an SQL database using a Python script. International Classification of Diseases (ICD)10 primary diagnoses, age, ethnicity and sex were obtained from specific hospital encounter numbers using an structured query language (SQL) query along with mortality data. A list of specific ICD10 codes of interest are included in the Supplementary Table 1.

### Sysmex XN analyzers

Whole blood collected in EDTA tubes were analyzed using XN-1000 and XN-3000 hematology analyzers (Sysmex). The XN-3000 is made up of two XN-1000 modules and produces the same hematology parameters. The instrument uses fluorescence flow cytometry, impedance, hydrodynamic focusing, cynaide-free sodium lauryl sulphate (SLS) for hemoglobin and is capable of processing up to 100 samples/h using 88 μl sample volume. Up to 38 clinical parameters and 50 research parameters, or derivatives thereof are produced with up to 23 scattergrams and 4 histograms. The instrument stores up to 100,000 records in a buffer. A glossary of hematology parameter acronyms and explanations are available in Supplementary Table 2.

### SARS-CoV-2 PCR testing

Respiratory pathogen PCR testing was performed at the clinical laboratory at WDHB using a BDMax (Becton, Dickinson and Company, NJ, USA). Testing included screening for SARS-CoV-2, influenza A, B and respiratory syncytial virus. Positive PCR results were used to identify cases whereas negative PCR results were considered controls. Untested patients were not used in ML models for COVID-19 as the infection could not be fully excluded in all patients due to shifting diagnostic and testing criteria. Some patients underwent repeated COVID-19 testing for either confirmation or exclusion of infection. PCR results were linked to hematology data via linkage through a laboratory information system.

### Prediction of age, sex & individuality

After excluding data with missing demographics ML models were developed for sex, age and individuality. The purpose of this was to demonstrate the power of hematology data to discriminate and predict objectives, using feature patterns that would otherwise have been indistinguishable to a human observer. These objectives are clearly definable but not outwardly clinically useful.

### Prediction of infectious diseases & COVID-19

Randomly selected first presentation FBCs from the total dataset were selected as controls for training models for both pneumonia and urinary tract infection. Only one FBC from each unique patient was used to ensure models did not train on features of individuality. Models were trained, tested and then validated in an independent cohort. Due to the low number of COVID-19 PCR-positive cases, serial results for each positive case were used for descriptive statistics and in ML models, in the assumption that this would include the various stages of the disease and convalescence. Hematology data from patients who had undergone a respiratory PCR test at North Shore Hospital’s XN-3000 was used for training with validation performed on data from Waitakere Hospital’s XN-1000. All data were pooled for binary prediction of a positive SARS-CoV-2 PCR result and for an identification of a specific virus in PCR-positive cases. Models were then applied to data from 9 June 2020 to 24 August 2020 during New Zealand's second wave.

### Prediction of heart failure

Heart failure was chosen as an example of a common noncommunicable disease, as it was the original objective of this project in July 2019. Due to the higher number of patients only single FBC results were used for ML using an approximate number of matched randomly selected controls. Multiple models were generated using either just hematology data or hematology data combined with age, demographics and standard laboratory biochemistry data and compared in an independent validation, inclusive of the remaining total dataset.

### Statistics & ML

Univariate analysis was performed using the student *t*-test for continuous parametric variables and receiver-operating characteristic curve analysis was used to assess performance of diagnostic biomarkers by c-statistic. All tests were two-tailed and p < 0.05 deemed statistically significant, except where Bonferroni correction for multiplicity was applied. Medcalc software version 16.8.4 was used to analyze the data. BigML https://bigml.com/ was used for applying ML models, using decision trees, and ensembles, logistic regression and deep neural networks (DNN) with transparency (https://static.bigml.com/pdf/BigML_Classification_and_Regression.pdf?ver=c306567#page=250). Model development involved splitting data 80:20 into training and test sets, and in the context of pneumonia, urinary tract infection and heart failure included an independent validation set. OptiML, an automated BigML optimization process for model selection and parametrization was used to find the best supervised model for sex classification and predicting age using regression. OptiML uses Bayesian parameter optimization and Monte Carlo cross-validation (https://static.bigml.com/pdf/BigML_OptiML.pdf?ver=c306567). Unsupervised ML included t-stochastic neighbor embedding (t-SNE) embedding projector https://projector.tensorflow.org/ applied to numeric CSV data and uniform manifold approximation and projection (UMAP) to visualize high dimensional flow cytometry FCS data.

### Data availability

The materials, data, code and associated protocols are available to readers with application to the corresponding author and Waitematā privacy, security and governance group with a limited data sharing agreement. BigML models will be shared without limitations.

## Results

A total of 156,570 FBCs were performed at both WDHB hospitals between 1 July 2019 and 8 June 2020 on 43,761 unique patients. A total of 65,227 of these FBC results had an ICD10 primary diagnosis which could be linked to an encounter. The primary diagnosis was unspecified pneumonia in 372, urinary tract infection in 229 and heart failure or cardiomyopathy in 375 unique patients. A maximum of 247 FBC features from CSV data were used; 134 categorical, 101 numeric.

Over this time period 9424 respiratory viral panels including 6129 SARS-CoV-2 PCR tests on 5625 individual patients were performed. A total of 2168 of these SARS-CoV-2 PCR tests could be matched to patients with FBC data. A total of nine patients with 102 FBCs were SARS-CoV-2 positive, 2159 patients with 15,243 FBCs were SARS-CoV-2 negative. Comparisons of hematology parameters across all FBCs are in Table 1 and biochemistry in Table 2. Summary results are available in Table 3.

**Table 1.** Hematology parameters compared between confirmed COVID-19 cases and controls.

Parameter	SARS-CoV PCR + n = 102	SARS-CoV-2 PCR − n = 15,244	*t*-test
Q-Flag (Left Shift?)	85.05	27.59	3.44E-22
(MO-X[ch])	126.86	123.11	9.74E-19
[HFLC%(%)]	0.82	0.20	1.58E-16
(NE-SFL [ch])	55.80	51.03	3.14E-16
Q-Flag (Atypical Lympho?)	76.14	29.38	7.75E-14
BASO#(10^9^/L)	0.03	0.05	5.8E-10
(LY-WX)	576.05	528.17	2.69E-09
(LY-Z[ch])	63.22	61.72	2.15E-08
BASO%(%)	0.33	0.51	6.09E-08
(HFLC#[10^9^/L])	0.05	0.02	8.35E-08
PCT (%)	0.31	0.25	1.5E-07
(BA-N%[%])	0.35	0.52	8.79E-07
(MO-WX)	242.90	261.11	9.58E-06
(PLT-I [10^9^/L])	302.25	251.33	2.85E-05
PLT (10^9^/L)	302.25	251.47	3.01E-05
(MO-WY)	636.07	691.92	4.2E-05
Q-Flag (Blasts/Abn Lympho?)	105.22	88.91	0.0002
RDW-CV (%)	14.06	14.99	0.0002
EO#(10^9^/L)	0.10	0.18	0.0003
EO%(%)	1.24	2.23	0.0003

**Table 2.** Biochemistry and standard parameters compared between confirmed COVID-19 cases and controls.

	SARS-CoV PCR + n = 37	SARS-CoV PCR − n = 6624	*t-*test
Albumin (g/l)	27.5	37.9	9.04E-10
Sodium (mmol/l)	141.6	139.1	1.16E-05
Hemoglobin (g/l)	119	133.2	1.85E-05
Lipase (U/l)	80	41	0.056

**Table 3.** Summary results of varying machine learning models.

	Model AUROC		
	Training	Test	Validation
Biological sex	0.84	0.83	-
Pneumonia	0.82	0.83	0.74
Urinary tract infection	0.81	0.84	0.68
Heart failure	0.91	0.91	0.78
COVID-19	0.98	0.99	0.8

AUROC: Area under the receiver operating curve.

### Prediction of age, biological age, sex & individuality

**Figure 1.**
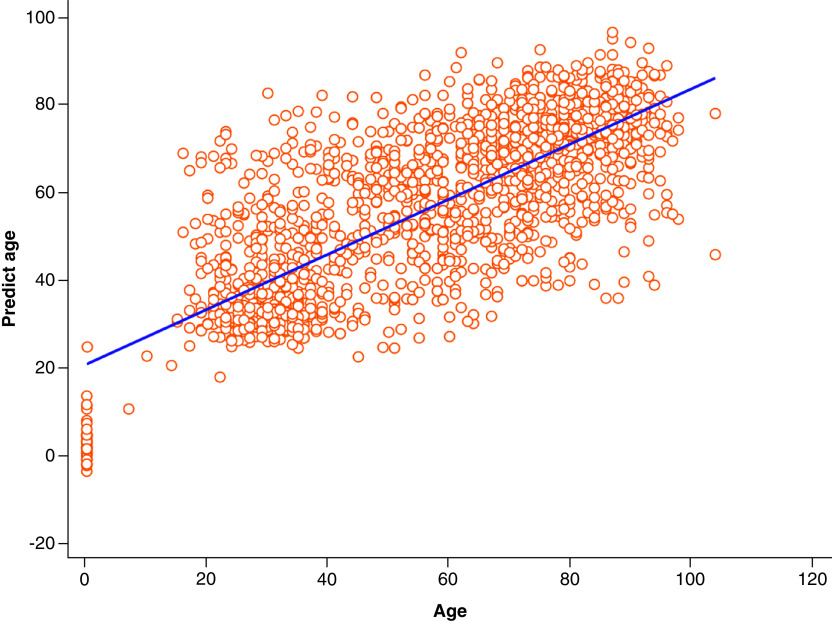
Scatterplot and regression line demonstrating deep neural networks predicted age versus chronological age.

Chronological age was available for 8462 unique patient FBCs. A DNN produced the highest R^2^ of 0.55 to predict age, with mean absolute error 12. Highest ranked features were [LY-Z(ch)], LY-X(ch), RDW-SD(fL), MONO%(%), LY-WY. This validated in the test set of 1717 patients with an R^2^ of 0.59, mean absolute error 12 (Figure 1).

Predictions of biological sex were made on single FBCs from 20,531 unique patients. A total of 11,917 (58%) were female, 8614 (42%) were male. A bootstrap decision forest predicted sex with an AUROC of 0.84, phi: 0.53 in the training set and was validated in the test set with AUROC: 0.83 and phi: 0.47 in 5051 unique patient FBCs. Highest ranked features were haemoglobin (HGB) (g/l), haematocrit (HCT) (ratio), MONO%(%), RBC (10^12^/L), (LY-X[ch]).

**Figure 2.**
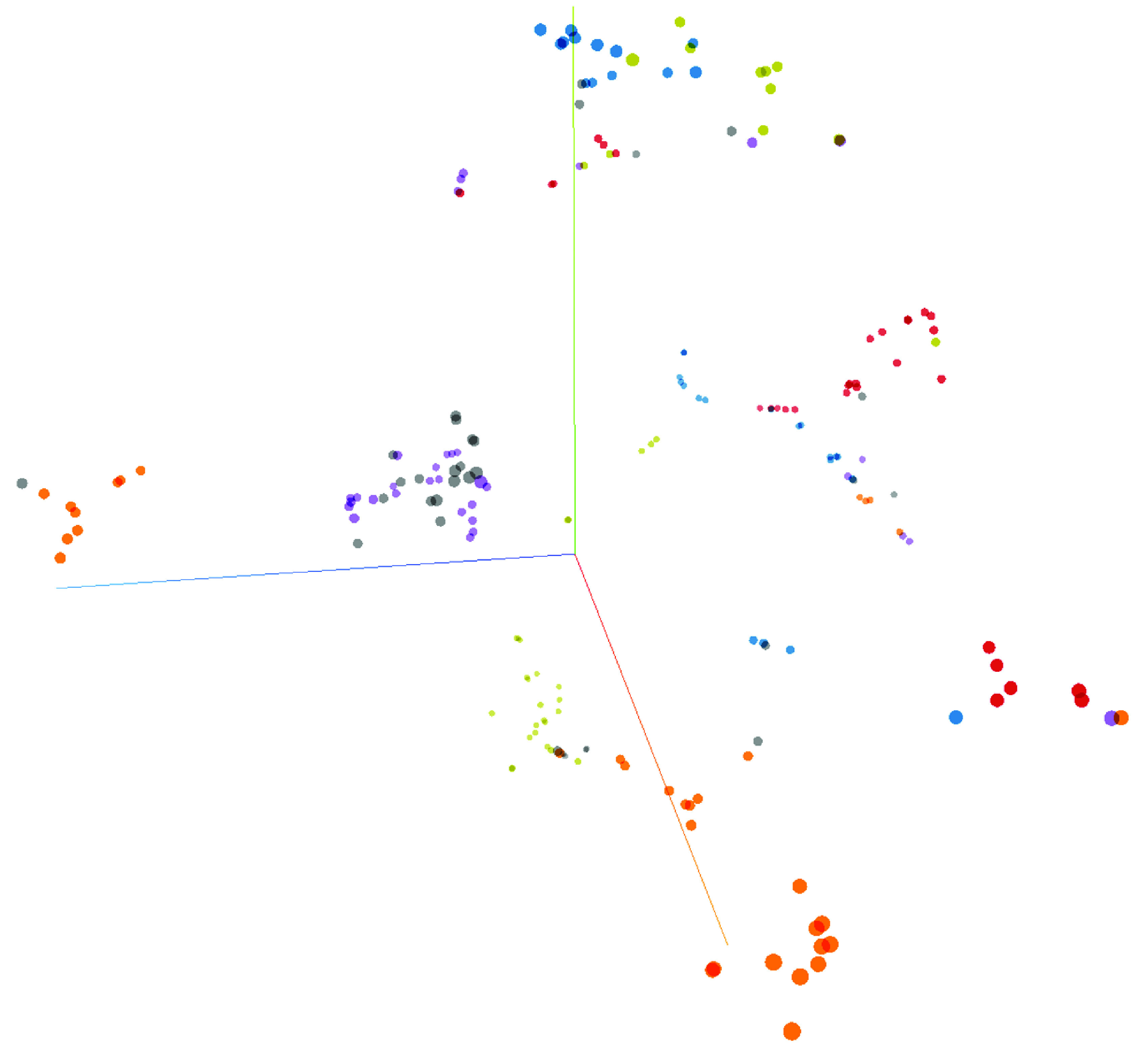
t-SNE (unstructured machine learning clustering) of six patients each undergoing ten full blood counts over time.

A total of 13 patients with more than 100 unique serial FBCs were selected from the complete dataset with an aim to predict individuality in FBC patterns. A DNN predicted an individual patient’s identity with 99.7% accuracy, F-measure: 0.97, precision: 100%, recall: 94.7% and phi: 0.97. The features used for this prediction varied according to ML method but MicroR (%) consistently ranked as the highest feature. To visualize the personalized nature of FBC metadata a t-SNE was created using only ten FBC results from six patients (Figure 2).

### Prediction of infectious diseases & COVID-19

A total of 250 patients with pneumonia and equal controls were used for model training and testing. A total of 122 pneumonia cases were used for independent validation in a dataset including 14,453 controls. A boosted decision tree returned an AUROC 0.82, phi: 0.58 in the training set; AUROC: 0.83, phi: 0.44 in the test set; which validated with an AUROC: 0.74, sensitivity: 58%, specificity: 79%, 95% CI: 0.73–0.75; p < 0.0001 (Supplementary Figure 1). Highest ranked features were NE-FSC(ch), MO-X(ch), LY-Z(ch), NE-SSC(ch) and LY-WY.

A total of 168 patients with urinary tract infection and equal controls were used for model training and testing. A total of 61 urinary tract infection cases were used for independent validation with 14,453 controls. A random decision forest returned an AUROC: 0.81, phi: 0.59 in the training set; AUROC: 0.84, phi: 0.41 in the test set; and validated with an AUROC: 0.68, sensitivity: 52%, specificity: 79%, 95% CI: 0.67–0.68; p < 0.0001. Highest ranked features were MO-X(ch), MO-WZ, PCT (%), RBC (10^12^/L) and LY-Z(ch)]. A logistic regression model designed to distinguish pneumonia from urinary tract infection returned an AUROC of 0.64, phi: 0.43 in the training set but AUROC: 0.84, phi: 0.46 in the test set.

**Figure 3.**
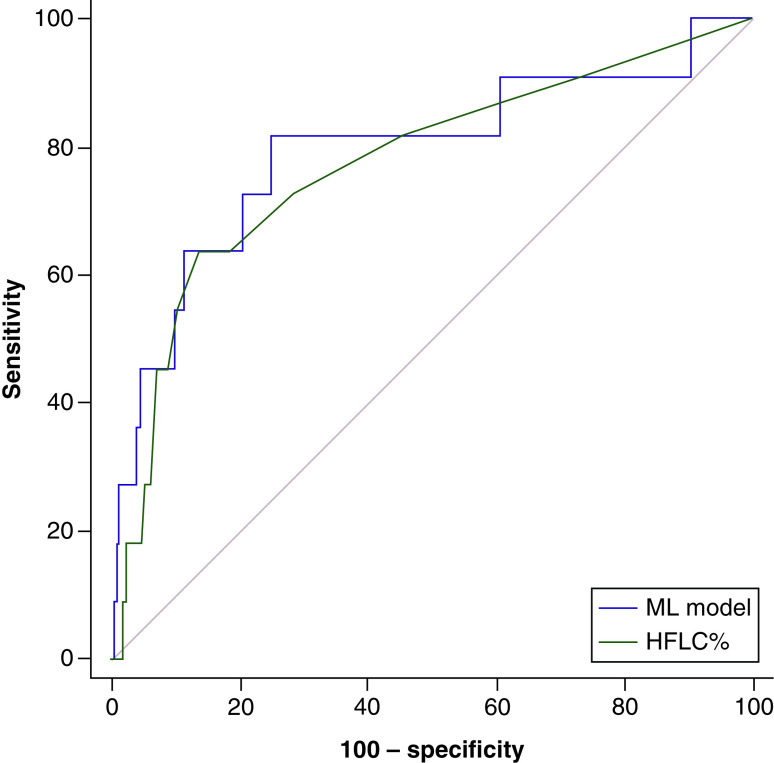
Comparison of univariate predictor HFLC%, a machine learning model for COVID-19. HFLC: Highly fluorescent lymphocytes count; ML: Machine learning.

A total of 102 instances (serial FBCs in nine patients identified during New Zealand’s first lockdown) and 204 control FBCs in unique patients were used for model training and testing. A total of 11 FBCs from three patients with COVID-19 were used for validation with 6770 controls. A boosted decision tree returned an AUROC: 0.98, phi: 0.96 in the training set and AUROC: 0.99, phi: 0.92 in the test set, which was most likely over-fitting however, in the independent validation set the model still exceeded the highest univariate predictor of COVID-19 (highly fluorescent lymphocytes count % [HFLC%] ), AUROC: 0.8, sensitivity: 82%, specificity: 75%; 95% CI: 0.79–0.8; p = 0.0006 (Figure 3) versus univariate AUROC: 0.77, though the difference was not statistically significant. Highest ranked features included Q-Flag (Abn Lympho?) and HFLC%. HFLCs originate from splenic marginal-zone B-cells and reflect high lymphocytic RNA transcription, IgM producing cells.

**Figure 4.**
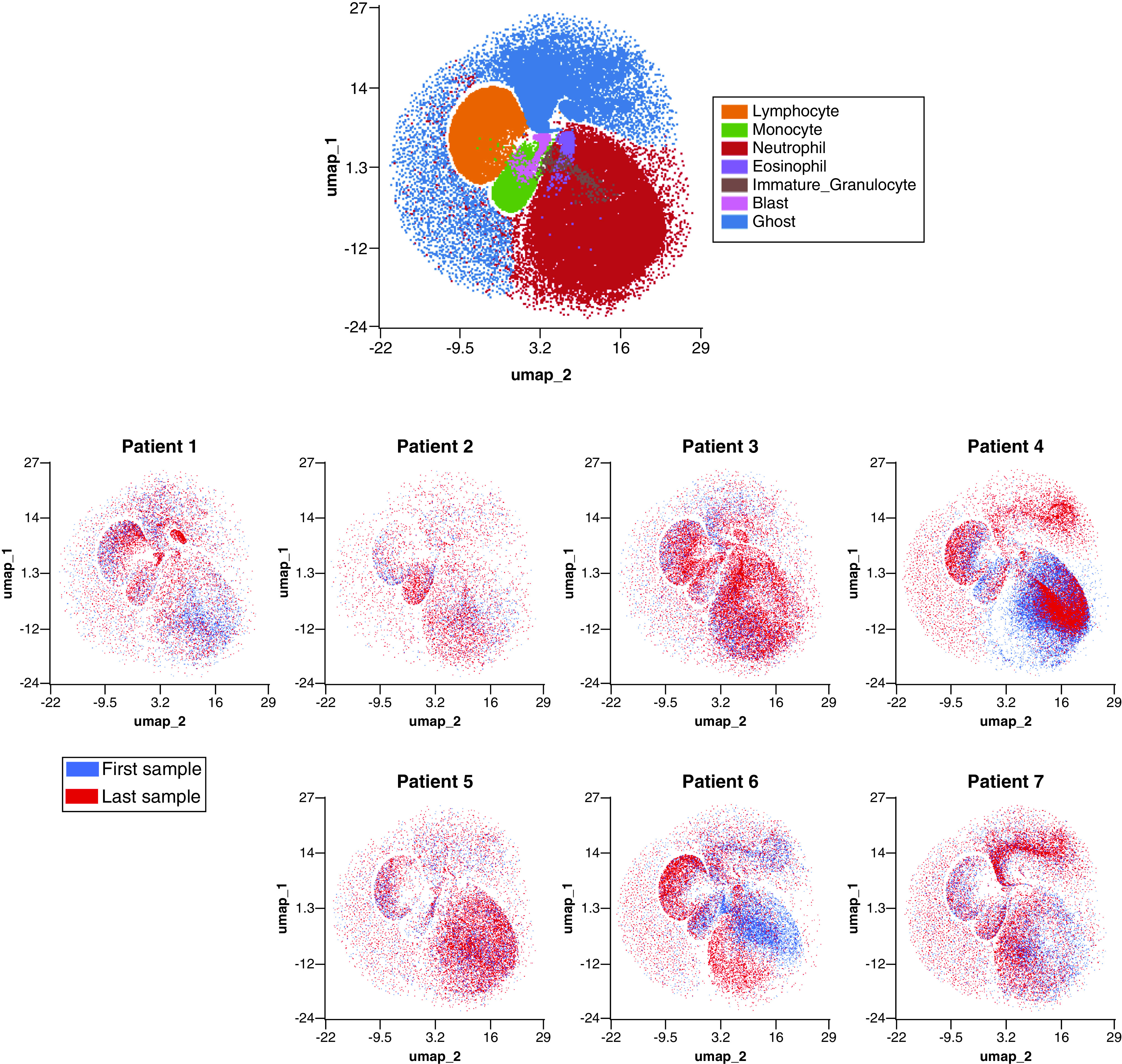
UMAP demonstrating highly individualized temporal cellular changes in COVID-19. Samples were processed in the OMIQ platform which allowed for metadata to be assigned to each sample. Samples were labeled by patient, time series point and whether the patient was deceased or not. Cells were initially manually gated based on the manufacturer’s guidelines for WDF samples to define lymphocytes (orange), monocytes (green), neutrophils (red), eosionphils (purple), immature granulocytes (brown) blasts (pink) and ghost/debris (blue). A UMAP analysis was performed using all available signal parameters (side scatter, side fluorescence, forward scatter and forward scatter pulse width). (Top) UMAP showing all cells colored by manually gated subsets. (Bottom) overlay UMAP plots of first (blue) and last (red) time points sampled from each of seven individual patients. Patients four and five were deceased. WDF: White cell differential; UMAP: Uniform manifold approximation and projection.

**Figure 5.**
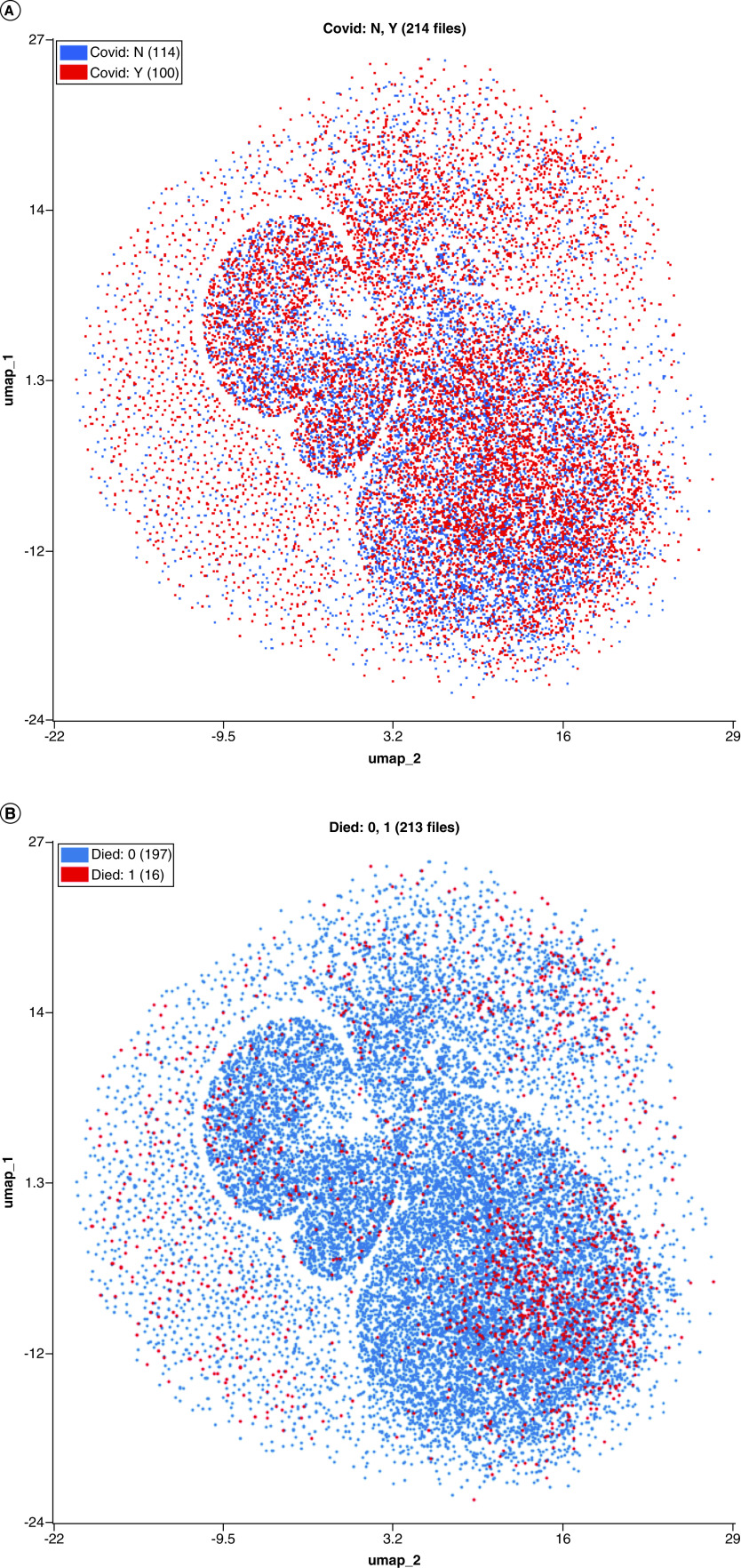
Discriminatory patterns between COVID-19 and non-COVID-19 patients and COVID-19 nonsurvivors. Samples were processed in the OMIQ platform which allowed for metadata to be assigned to each sample. Samples were labeled by patient, time series point and whether the patient was deceased or not. Cells were initially manually gated based on the manufacturer’s guidelines for WDF sample. A UMAP analysis was performed using all available signal parameters (side scatter, side fluorescence, forward scatter and forward scatter pulse width). Equal downsampling was performed to make plots interpretable with overlap. (Top) overlay UMAP showing cells from all samples colored by COVID-19 status. Blue cells indicate COVID-19 negative, red cells indicate COVID-19 positive. (Bottom) overlay UMAP showing cells from all samples colored by survival. Blue cells indicate survival, red cells indicate death due to COVID-19. UMAP: Uniform manifold approximation and projection.

UMAP visualizations were created to demonstrate the differences between COVID-19 and non-COVID-19 patients as well as trajectories in cellular response over time and outcome (Figures 4 & 5). UMAP parameters were generated using all four available parameters in the FCS files.

**Figure 6.**
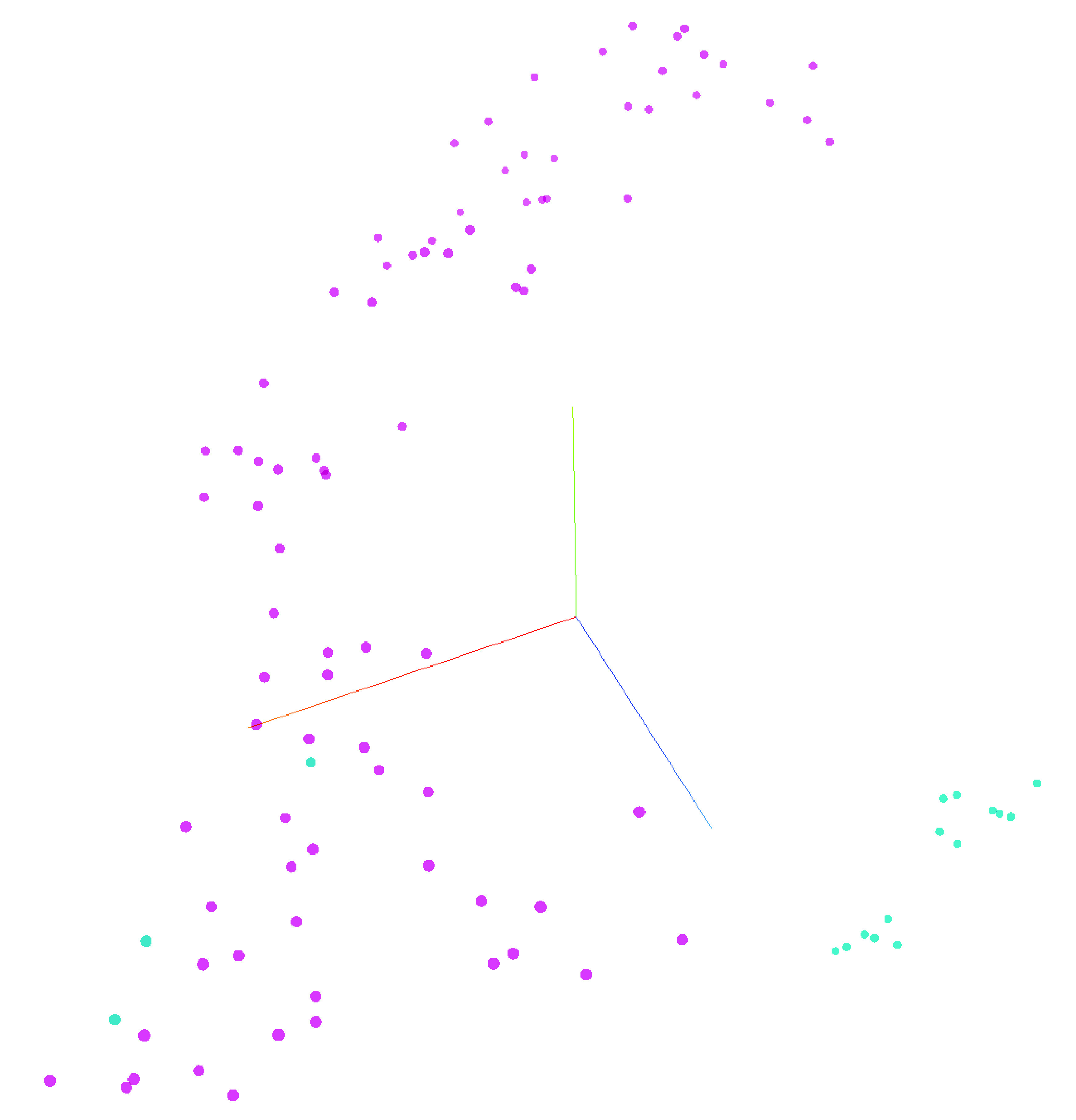
tSNE demonstrating temporal patterns in numeric hematology data between patients. Nonsurvivors (n = 2) (light blue) versus survivors (n = 7) (purple).

UMAPs were overlaid with recognizable population labels (lymphocyte, monocyte, neutrophil, immature granulocyte, blast/aberrant lymphocyte and ghost/debris) defined by manual gating (as per Sysmex guidelines) to aid in interpretation (Figure 4, top). Cells from each patient’s first (blue) and last (red) time point sampled were plotted together to determine shifts in cellular population characteristics over the course of disease (Figure 4, bottom). In all patients, there was a noticeable shift in the population characteristics between first and last time point. In most instances, these differences were within immune cell subsets (e.g., a change in neutrophil phenotype) rather than a change in the overall immune signature (e.g., a change in the ratio of neutrophils to monocytes). tSNE was used to visualize temporal changes and clustering of numeric hematology data, comparing survivors with nonsurvivors (Figure 6).

### Prediction of heart failure

A total of 237 heart failure patients and 384 controls were used for training and testing models for heart failure and cardiomyopathy. Models including age, sex and ethnicity negligibly differed from models excluding these demographics, so, to aid reproducibility these data were excluded. A logistic regression model returned an AUROC: 0.91, phi: 0.72 in training and AUROC: 0.91, phi: 0.62 in the test set. In the validation set of 138 cases and 42,615 controls the AUROC was 0.78, sensitivity of 72%, specificity of 72%, 95% CI: 0.77–0.78; p < 0.0001 (Supplementary Figure 2). Highest ranked features were RDW-SD(fL), PCT/M, BA-D#(10∧9/L), LY-WY, LY-Z(ch).

## Discussion

**Figure 7.**
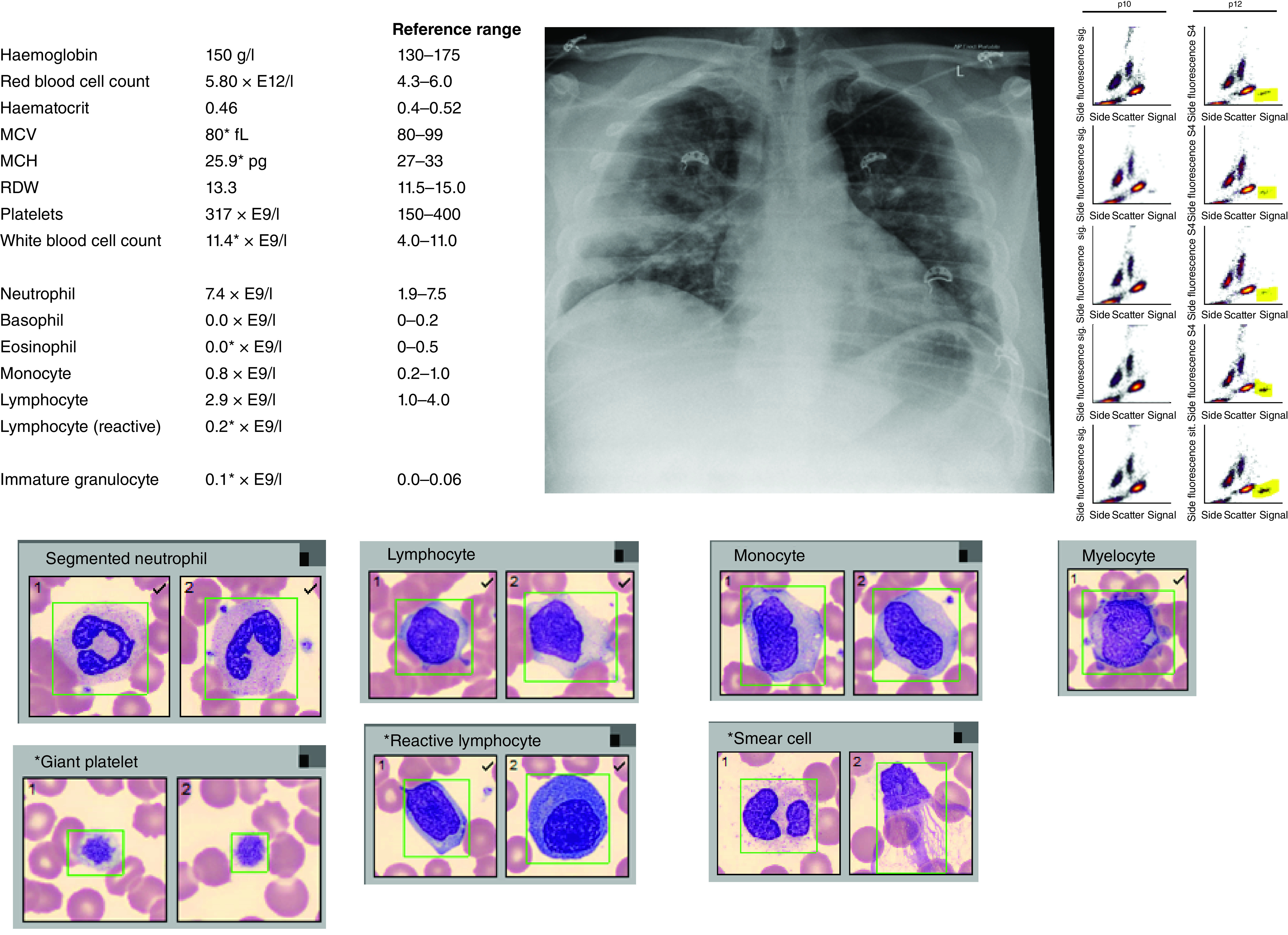
Full blood count data (upper left), chest x-ray findings (upper middle) and flow cytometry showing eosinopenia in a young man with COVID-19 pneumonia. Cellavision images (bottom) show unremarkable white cell morphology apart from reactive lymphocytes. Yellow highlighted region on scattergram (right) identifies eosinophils region, which were near absent in this patient. Asterix identifies lab result outside of the reference range. MCH: mean corpuscular hemoglobin; MCV: Mean corpuscular volume; RDW: Red cell distribution width.

In this paper, we aimed to demonstrate the hidden power of hematology metadata derived from a standard FBC. We have shown the ability to predict age, sex, individuality with a high degree of accuracy. Similarly, imperceptible patterns within hematology data (Figure 7) allowed the prediction of infectious diseases such as COVID-19, pneumonia and urinary tract infection as well as noncommunicable diseases such as heart failure. Although important, these predictions may not currently be sufficient to have clinical utility. However, with larger datasets spanning a wider breadth of pathophysiology, there may be an opportunity to improve upon these predictions. There are numerous examples where similar approaches, using standard laboratory data, have been used to predict the presence of COVID-19 with a relatively high degree of accuracy [12,16–18,26–33]. We used here only hematology data, demonstrating individual variables such as HFLC, previously associated with COVID-19 [34,35].

**Figure 8.**
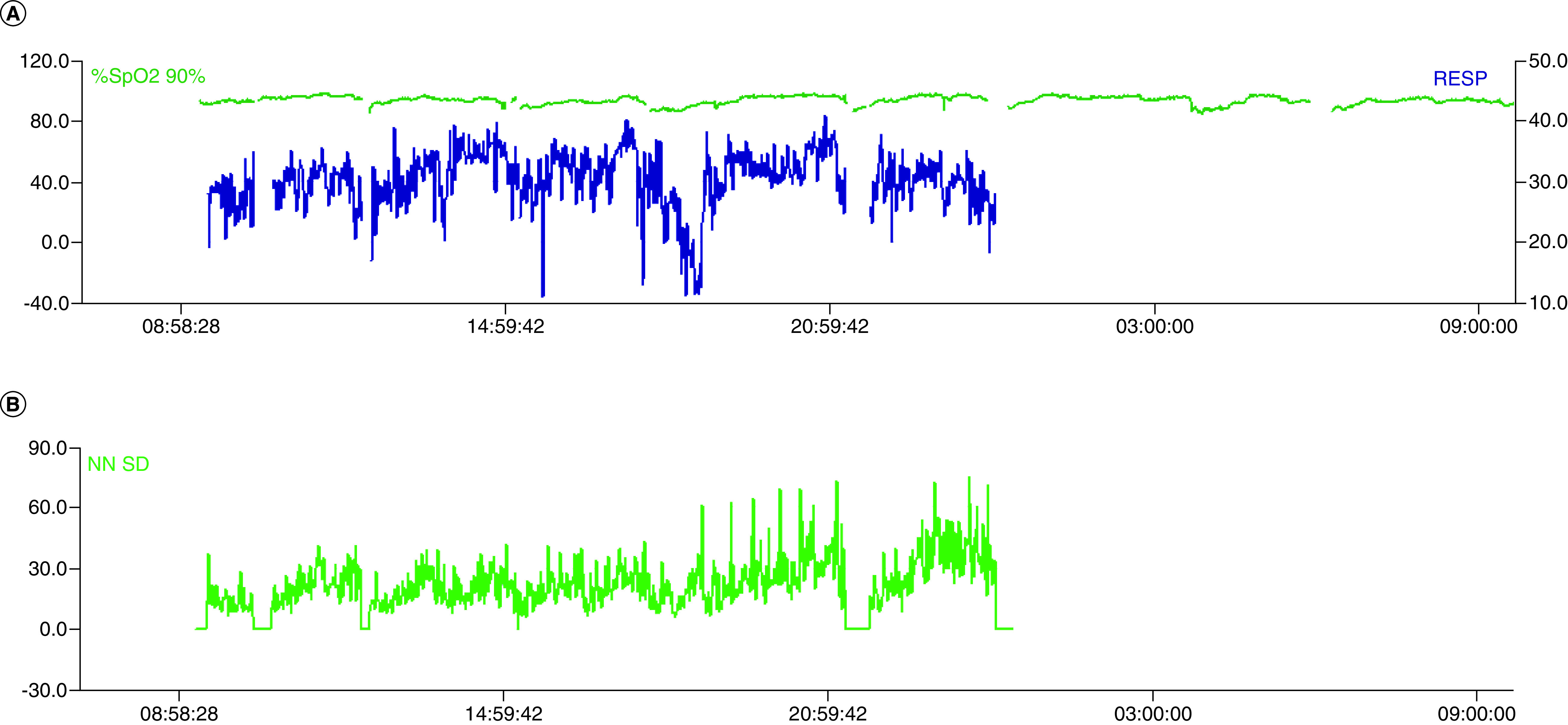
Heart rate and oxygen saturation tracings on a single patient with COVID-19 in a high dependency unit (top) with accompanying heart rate variability tracing (bottom).

ML applied to larger datasets of COVID-19 cases would likely provide the ability to prognosticate as well as diagnose. In a study by Soltan *et al.* ML applied to laboratory data and e-vitals, inclusive of temporal trajectories, were validated both retrospectively and prospectively with a reported accuracy of 92.5% [15]. Thanks to the digital infrastructure at Waitematā District Health Board e-vitals are captured digitally, giving us the capacity to include this data in future model iterations. Anecdotally, we have seen altered sleep/wake cycles, overnight hypoxia and abnormal heart rate variability in individual cases of COVID-19 (Figure 8). Perturbations in these variables, known to be influenced by pathogens [36], is probably what has been identified in the DETECT study which used wearable devices to identify influenza-like illness [37]. This also appears to be translatable to COVID-19 [33,38].

Blood is a rich source of information, and this study demonstrates that rapidly accessible inexpensive data that is otherwise purged can be put to new uses. Although this method, applied as a screening tool to 10,000’s of FBCs will generate false positives, it could be deployed with an automated alert or trigger downstream confirmatory lab tests, for example NT-proBNP for heart failure, metagenomic sequencing etc. Other emerging high throughput ‘omic’ technologies, such as metabolomics and ML, have been used to both predict the presence of COVID-19 with a high degree of accuracy and the [39] severity of pneumonia. This has only been achievable using the shared resource of UK Biobank [26]. Host metabolomic profiling could be achievable in New Zealand which would facilitate screening the population for targeted isolation or prioritized vaccination [40]. Sysmex hematology analyzers utilize flow cytometry to produce not only extracted numeric data, but also high dimensional data contained within FCS files similar to other ‘omic technologies. Numerous flow cytometry studies have demonstrated the utility of this method in identifying host responses to COVID-19. There appear to be individual immunophenotypes which are likely to predict not only prognosis but also response to treatment [41].

Artificial intelligence has been used in numerous ways to combat COVID-19, including not only the use of blood-based laboratory data but also radiomics and remote monitoring devices [42]. The hidden potential of these tools should be explored in both other communicable and noncommunicable diseases as they are likely to provide unexpected long-term benefits for numerous other health conditions, for example, cardiacvascular disease and heart failure [43]. For instance, a study using ML applied to longitudinal FBC data showed high accuracy in predicting the presence of bowel cancer which has been translated into a clinical tool [44]. Integrating other sources of highly abundant laboratory data with hematology data have been used to predict age or ‘biological age’ [45] also known as phenotypic age, which itself is associated with poorer outcomes in COVID-19 [46]. Integrating other forms of non laboratory data are likely to improve upon these predictions and we have previously shown that integrating conventional ECG parameters with laboratory data predicts the presence of heart failure with a high degree of accuracy (AUC: 0.94, sensitivity: 74%, specificity: 94%, phi: 0.58) [47].

**Figure 9.**
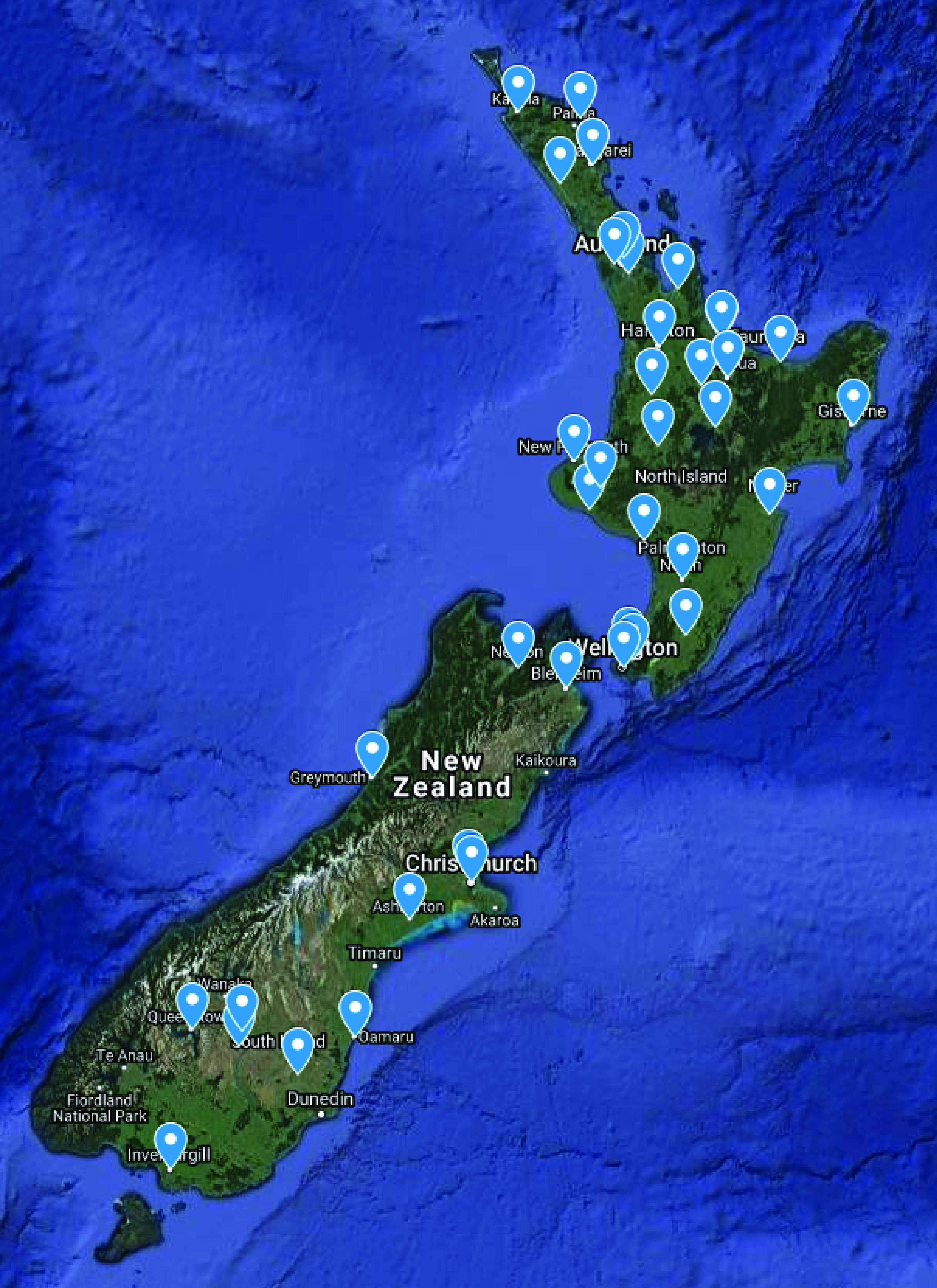
Sysmex hematology analyzer locations in New Zealand.

New Zealand's digital infrastructure has many advantages for the application of this technology. First, for the most part, it has embraced digital medicine and data are abundant, second, the population is ethnically diverse, third, ICD10 and outcome data are centralized and there is excellent longitudinal data capture. Testsafe electronic laboratory results, available for over a decade, has not only revolutionized access to blood results by both medical professionals and patients but it has been collecting and storing data for well over a decade. Moreover, Sysmex hematology analyzers have an 85% market share in New Zealand (Figure 9), meaning that by connecting systems together could increase the power of the network exponentially (Metcalfe’s law). In such a national, or even international system the ML methods described here would not only benefit individual clinicians and hospitals but could also provide real time assessment of populations for public health planning during pandemics. Since flow cytometry involves the profiling of immune cells, networked hematology analyzer systems linked to viral genomic sequences could geographically map the host responses to SARS-CoV-2 viral clades as they emerge. Such a system could be used in geographic-based outbreak analytics visualizations or vaccine programs in near real-time.

However, what cannot be overstated is appreciating the source of these data, which is the population of New Zealand. The data used in this project were obtained with ethical approval but without informed consent, due to the impracticality of obtaining it at scale. Secondary use of medical data carries significant issues around the maintenance of security and privacy of individuals. We have shown here that a simple FBC is highly personalized and acts as a fingerprint for each patient. Data sovereignty particularly for indigenous populations, in this context Māori, must be respected. Governance and kaitiakitanga is paramount. Establishing a social license for the secondary use of unconsented health data is also necessary [48]. Ideally data of this nature would be pooled and shared both locally and globally to deliver the scale needed for robust applications of artificial intelligence, however, there are governance, privacy and other issues to address first before this can be achieved. As hematology analyzers are ubiquitous across New Zealand, the accessibility of this technology, even in rural areas is high. As flow cytometry becomes portable it would be expected that these results will be translatable to handheld systems, further improving access to remote locations. Ultimately, what we have described here is just the beginning of a machine learning program, and there is significant work to be done to ensure the systems described are robust. To support this, government funders will need to build on the existing sparse infrastructure to support both personalized and genomic services in New Zealand, so that its benefits can be realized.

## Limitations

The sample of patients with COVID-19 in this study was too small to generate robust ML models, therefore, all FBC results on individual patients were used. This would have led to overfitting in model training with the predictive model identifying the individual patient, perhaps more than the disease. As we used transparent, explainable artificial intelligence (AI) with BigML we were able to interrogate the features used in predictive models. The COVID-19 data used here would also have been skewed toward those who were the most unwell with the infection and had a large number of blood tests. Most COVID-19 patients were managed at home and would not have undergone an FBC, therefore, the results here, do not represent the entire spectrum of disease. Although ML models may have over fitted with reduced availability of data, the data used for pneumonia and heart failure models, generated on unique patient data, were robust. FCS data were not available on all patients as the time taken to download it from the IPU made it impractical to obtain. ICD10 diagnoses are reliant on accurate coding and not always reliable, missing data limited the number of patients used in statistical models. Modest predictions are not clinically useful in the context of a disease at low prevalence, with both low positive and negative predictive value. To evaluate the clinical utility of the method outlined in this project, ML predictions would need to show incremental benefit to standard of care with a history, physical examination and conventional investigations. RT-PCR was used as the gold standard, but false negatives may have occurred resulting in misclassification of patients.

## Future perspective

ML/artificial intelligence will have many applications in medicine. ML predictions made from laboratory data are an excellent example where existing, inexpensive data are converted to more valuable information. Understanding how this will impact and change clinical practice will be important in the future as the implications of implementing a machine learning tool at scale could be profound. These implications could be positive or negative depending on the downstream effects on resource utilization and clinical outcomes. Laboratory data including raw machine outputs are an untapped resource, rich in both snapshot and time series health information. Future research in this field will require pooling data to generate adequately sized datasets of rarer diseases, and careful clinical implementation studies to gauge the impact on physician behavior and patient outcomes.

**Summary points**

- We undertook a machine learning project, making use of existing hematology analyzer raw data, much of which is purged from healthcare systems.
- These data were rich in information about health states and predicted biological age, gender, individuality and both communicable and noncommunicable diseases, such as COVID-19 and heart failure.
- Further work will be required to evaluate the clinical impact of these machine learning tools.
