# Supplementary material for: A machine learning PROGRAM to identify COVID-19 and other diseases from hematology data
Source: Future Sci OA. 2021 Jun 12;7(7):FSO733. doi: 10.2144/fsoa-2020-0207 (PMC8204819; doi:10.2144/fsoa-2020-0207)

Table 1. ICD10 codes used in PROGRAM

| **ICD Code** | **ICD full desc** |
| --- | --- |
| **A390** | Meningococcal meningitis |
| **A394** | Meningococcaemia, unspecified |
| **A399** | Meningococcal infection, unspecified |
| **A419** | Sepsis, unspecified |
| **A870** | Enteroviral meningitis |
| **A879** | Viral meningitis, unspecified |
| **B342** | Coronavirus infection, unspecified site |
| **B972** | Coronavirus as the cause of diseases classified to other chapters |
| **G001** | Pneumococcal meningitis |
| **G002** | Streptococcal meningitis |
| **G008** | Other bacterial meningitis |
| **G009** | Bacterial meningitis, unspecified |
| **J100** | Influenza with pneumonia, other influenza virus identified |
| **J101** | Influenza with other respiratory manifestations, other influenza virus identified |
| **J108** | Influenza with other manifestations, other influenza virus identified |
| **J110** | Influenza with pneumonia, virus not identified |
| **J111** | Influenza with other respiratory manifestations, virus not identified |
| **J118** | Influenza with other manifestations, virus not identified |
| **J120** | Adenoviral pneumonia |
| **J121** | Respiratory syncytial virus pneumonia |
| **J122** | Parainfluenza virus pneumonia |
| **J123** | Human metapneumovirus pneumonia |
| **J128** | Other viral pneumonia |
| **J129** | Viral pneumonia, unspecified |
| **J13** | Pneumonia due to Streptococcus pneumoniae |
| **J150** | Pneumonia due to Klebsiella pneumoniae |
| **J151** | Pneumonia due to Pseudomonas |
| **J152** | Pneumonia due to staphylococcus |
| **J154** | Pneumonia due to other streptococci |
| **J155** | Pneumonia due to Escherichia coli |
| **J156** | Pneumonia due to other (aerobic) Gram-negative bacteria |
| **J157** | Pneumonia due to Mycoplasma pneumoniae |
| **J158** | Other bacterial pneumonia |
| **J159** | Bacterial pneumonia, unspecified |
| **J180** | Bronchopneumonia, unspecified |
| **J181** | Lobar pneumonia, unspecified |
| **J182** | Hypostatic pneumonia, unspecified |
| **J188** | Other pneumonia, organism unspecified |
| **J189** | Pneumonia, unspecified |
| **J81** | Pulmonary oedema |
| **N390** | Urinary tract infection, site not specified |
| **U072** | Emergency use of U072 |
| **I500** | Congestive heart failure |
| **I509** | Heart failure, unspecified |
| **I130** | Hypertensive heart and kidney disease with (congestive) heart failure |
| **I110** | Hypertensive heart disease with (congestive) heart failure |
| **I132** | Hypertensive heart and kidney disease with both (congestive) heart failure and kidney failure |

Table 2. Sysmex haematology parameters

| **Parameter** |  | **Technical definition** |
| --- | --- | --- |
| BA-D#(10^9/L) | Basophil counts from WDF channel | The basophil counts calculated from the WDF channel |
| [BA-N%(%)] | Basophil count in % | Basophil count from the WNR scatter |
| BASO#(10^9/L) | Basophil count (absolute) |  |
| BASO%(%) | Basophil count (in %) |  |
| [HFLC#(10^9/L)] | High Fluorescence lymphocytes count in absolute | The count of the upper LYMPH area of the WDF scattergram |
| [HFLC%(%)] | High Fluorescence lymphocytes count | The ratio of the count of the upper LYMPH area of the WDF scattergram to the WBC count |
| EO#(10^9/L) | Eosinophil count (Absolute) |  |
| EO%(%) | Eosinophil count (%) |  |
| LY-X(ch) | Lymphocyte scatter | The lateral scattered light intensity of the LYMPH area of the WDF scattergram |
| [LY-Z(ch)] | Lymphocyte scatter | The forward scattered light intensity of the LYMPH area on the WDF scattergram |
| [LY-WX] | Lymphocyte scatter | The lateral scattered light distribution width of the LYMPH area on the WDF scattergram |
| LY-WY | Lymphocyte scatter | The Fluorescence light distribution width of the LYMPH area on the WDF scattergram |
| MONO% | Proportion of monocyte in sample | Monocyte percent |
| [MO-X(ch)] | Monocyte scatter | The lateral scattered light intensity of the MONO area on the WDF scattergram. |
| [MO-WX] | Monocyte scatter | The lateral scattered light distribution width of the MONO area on the WDF scattergram |
| [MO-WY] | Monocyte scatter | The fluorescent light distribution width of the MONO area on the WDF scattergram. |
| MO-WZ | Monocyte scatter | The forward scattered light distribution width of the MONO area on the WDF scattergram |
| MicroR% | Proportion of microcytic red | Micro RBC ratio |
| NE-FSC(ch) | Neutrophil- Forward Scatter | The forward scattered light intensity of the NEUT area on the WDF scattergram |
| NE-SSC(ch) | Neutrophil- Side Scatter | The lateral scattered light intensity of the NEUT area on the WDF scattergram |
| [NE-SFL(ch)] | Neutrophil- Side Fluorescence | The fluorescent light intensity of the NEUT area on the WDF scattergram. |
| PCT(%) | Platelet Crit in % | Volume occupied by platelets in the blood https://www.ncbi.nlm.nih.gov/pmc/articles/PMC4910273/ |
| Q-Flag (Blasts/Abn Lympho?) | See explanation below* |  |
| PLT(10^9/L) | Platelet count |  |
| Q-Flag (Left Shift?) | See explanation below* |  |
| [PLT-I(10^9/L)] | Platelet Count_ Impedance |  |
| Q-Flag(Atypical Lympho?) | See explanation below* |  |
| RBC(10^12/L) | Absolute red blood cell count in sample | Red blood cell (erythrocyte) count |
| RDW-CV(%) | Red cell distribution width in CV | Indicates RBC variation in size (volume) |
| RDW-SD (fL) | Red cell distribution width (SD) | Red Cell Distribution Width (Standard Deviation) |

Parameter definition: <http://www.annlabmed.org/journal/view.html?uid=2758&vmd=Full>

*Q-flags is a built-in flagging algorithm of the XN series which indicates probability of an abnormal cells it is flagged for and according to the flag threshold value. Default is set at 100 (but adjustable). A Q-flag of ≥ 100 indicates a positive.

Figure 1. AUROC for boosted decision tree model to predict pneumonia from FBC


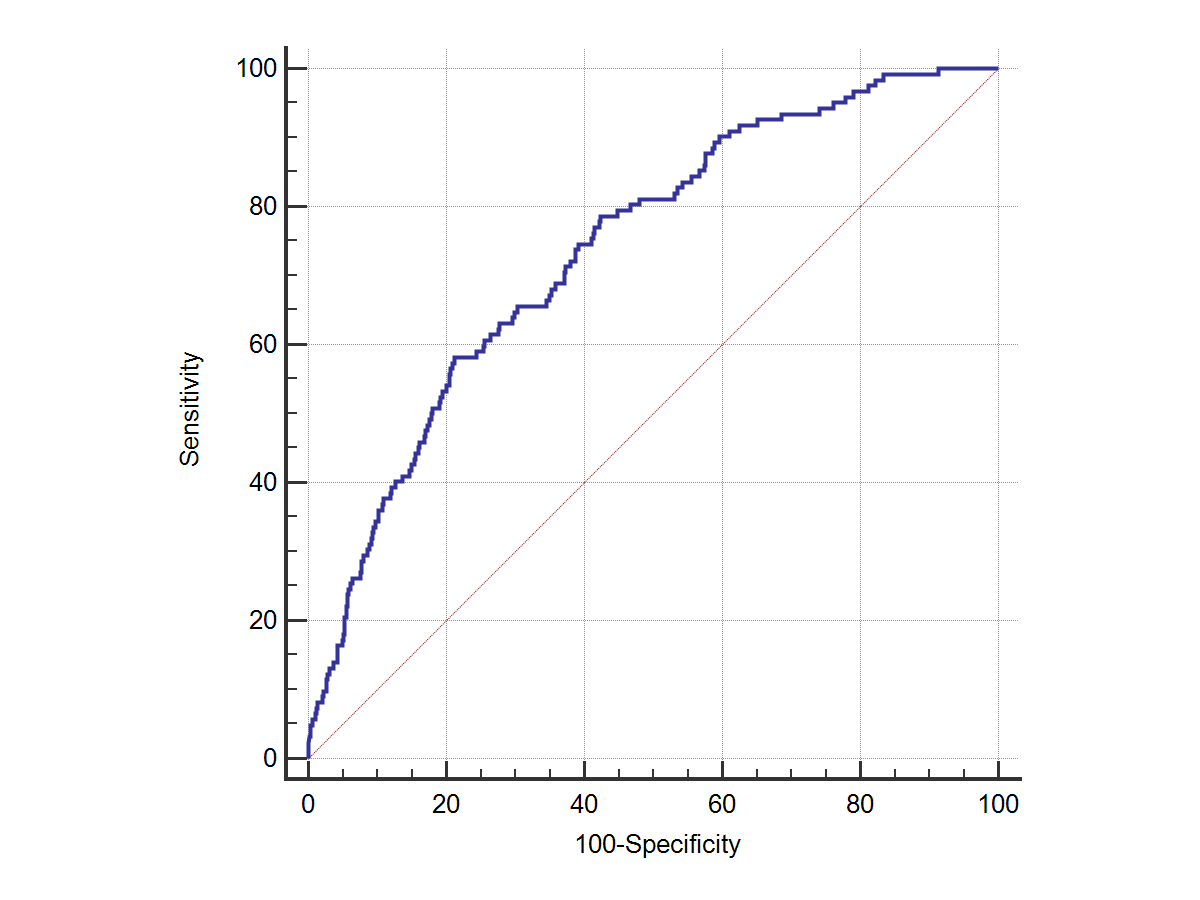


Figure 2. AUROC for logistic regression model to predict heart failure from FBC


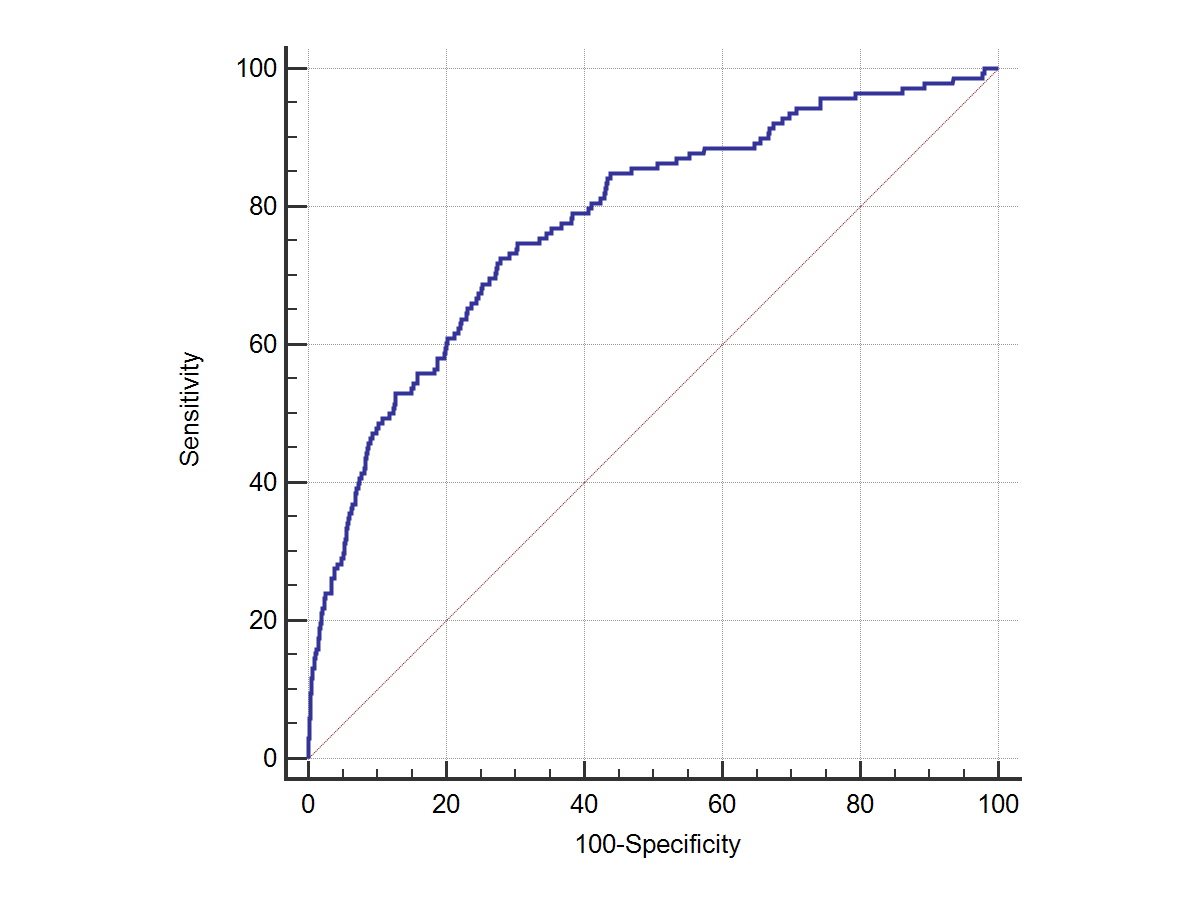

Supplement: Supplementary file 1 [file fsoa-07-733-s1.docx]
